# Asymmetric Distributions of Achromatic Bipolar Cells in the Mouse Retina

**DOI:** 10.3389/fnana.2021.786142

**Published:** 2022-01-13

**Authors:** Zachary J. Sharpe, Angela Shehu, Tomomi Ichinose

**Affiliations:** Department of Ophthalmology, Visual and Anatomical Sciences, Wayne State University School of Medicine, Detroit, MI, United States

**Keywords:** retina, GFP, immunohistochemistry, bipolar cells, topography

## Abstract

In the retina, evolutionary changes can be traced in the topography of photoreceptors. The shape of the visual streak depends on the height of the animal and its habitat, namely, woods, prairies, or mountains. Also, the distribution of distinct wavelength-sensitive cones is unique to each animal. For example, UV and green cones reside in the ventral and dorsal regions in the mouse retina, respectively, whereas in the rat retina these cones are homogeneously distributed. In contrast with the abundant investigation on the distribution of photoreceptors and the third-order neurons, the distribution of bipolar cells has not been well understood. We utilized two enhanced green fluorescent protein (EGFP) mouse lines, Lhx4-EGFP (Lhx4) and 6030405A18Rik-EGFP (Rik), to examine the topographic distributions of bipolar cells in the retina. First, we characterized their GFP-expressing cells using type-specific markers. We found that GFP was expressed by type 2, type 3a, and type 6 bipolar cells in the Rik mice and by type 3b, type 4, and type 5 bipolar cells in the Lhx4 mice. All these types are achromatic. Then, we examined the distributions of bipolar cells in the four cardinal directions and three different eccentricities of the retinal tissue. In the Rik mice, GFP-expressing bipolar cells were more highly observed in the nasal region than those in the temporal retina. The number of GFP cells was not different along with the ventral-dorsal axis. In contrast, in the Lhx4 mice, GFP-expressing cells occurred at a higher density in the ventral region than in the dorsal retina. However, no difference was observed along the nasal-temporal axis. Furthermore, we examined which type of bipolar cells contributed to the asymmetric distributions in the Rik mice. We found that type 3a bipolar cells occurred at a higher density in the temporal region, whereas type 6 bipolar cells were denser in the nasal region. The asymmetricity of these bipolar cells shaped the uneven distribution of the GFP cells in the Rik mice. In conclusion, we found that a subset of achromatic bipolar cells is asymmetrically distributed in the mouse retina, suggesting their unique roles in achromatic visual processing.

## Introduction

The eyes of vertebrates contain a single lens and retina, which evolutionarily split from the compound eyes of arthropods millions of years ago (Peterson et al., 2004; Jones, 2014). Since the separation, the structure of the retinas of vertebrates has evolved similarly among species, from fish to primates. The structure consists of five major neurons: photoreceptors, bipolar cells, ganglion cells, horizontal cells, and amacrine cells (Kolb, 2011). However, adaptational changes are recognized among animals with different body heights and habitats through the distinct topographies of retinal neurons. The topography of photoreceptors has been well investigated among species. Most non-primate mammals have two types of cones: short-wavelength sensitive cones (S-cones) and middle/long-wavelength sensitive cones (M/L-cones). However, some rodents lack S-cones, potentially due to their nocturnality (Peichl and Moutairou, 1998). Furthermore, the distribution of M- and S-cones in the retina is diverse depending on the height and habitat of the animals. The shape of the visual streak is longitudinal along the nasal-temporal axis for short-height animals, while the visual streak of taller animals exhibits dorsal extension (Peichl, 2005; Schiviz et al., 2008).

In the mouse retina, two types of photoreceptors, S- and M-cones, are heterogeneously distributed in the ventral and dorsal retinas, respectively (Szel et al., 1992). The inhomogeneous distributions of the two cones suggest that S-cones in the ventral retina enable the mouse to look up at the sky and M-cones in the dorsal regions support looking down at the grass. However, the dual opsin-expressing cones exist in the ventral retina (Haverkamp et al., 2005), and the color recognition of these two regions appears to be more complicated (Denman et al., 2018). Color vision by two wavelength-opponency primarily occurs in the ventral retina, which supports the upper visual field, by involving rod photoreceptors (Szatko et al., 2020) or by concentrated S-cones in the ventral retina (Nadal-Nicolas et al., 2020).

In addition to photoreceptors, the heterogeneous distributions of other retinal neurons have been gradually reported in the mouse retina. The M1-type melanopsin ganglion cells are asymmetrically distributed along the ventral-dorsal axis (Sondereker et al., 2017). Furthermore, OFF-alpha transient ganglion cells (OFF-α T RGCs) exhibit functional heterogeneous distributions; their light-evoked responses are transient in the ventral retina but are sustained in the dorsal retina (Warwick et al., 2018). This might be attributed to a distinct inhibitory network (Warwick et al., 2018) or the distinct locations and lengths of the initial segment of OFF-α T RGCs in two different retinal regions (Werginz et al., 2020).

Bipolar cells consist of approximately 15 types of chromatic and achromatic cells (Wässle et al., 2009; Breuninger et al., 2011). Type-dependent, diverse functional architecture is suggested for achromatic cells, such as motion detection (Euler et al., 2014; Hellmer et al., 2021). Bipolar cell topography was investigated by Camerino et al. (2021), which revealed that many types of OFF bipolar cells occurred at a higher density in the ventral retina than in the dorsal retina. However, the topography of other types of bipolar cells has not been investigated. To examine the topography of bipolar cells, we used two mouse lines that express the green fluorescent protein (GFP) in some types of bipolar cells. We found a heterogeneous distribution of some types of bipolar cells in the retina, suggesting their unique functions.

## Materials and Methods

### Ethical Approval

All animal procedures were approved by the Institutional Animal Care and Use Committee at the Wayne State University (protocol no. 20-10-2909). All the necessary steps were taken to minimize animal suffering. The tissues were harvested immediately after the animal was euthanized by CO_2_ inhalation and cervical dislocation.

### Mice

The 6030405A18Rik-EGFP (RRID:MMRRC_030515-UCD) and Lhx4-EGFP (RRID:MMRRC_030699-UCD) mouse lines, henceforth referred to as Rik and Lhx4, respectively, were obtained. The Rik line expresses enhanced green fluorescent protein (EGFP) under the control of the serine-rich and transmembrane domain-containing 1 (*Sertm1*) promoter. The Lhx4 line expresses GFP under the control of the LIM homeobox protein 4 (*Lhx4*) promoter. After they were received from the MMRRC, mice were crossed with C57BL6/J mice (Jackson Lab, Stock #000664) for more than 5 generations before being used for this study. Mice were maintained on a 12-h light/dark cycle and provided with free access to food and water. Litters were routinely genotyped for transgenes using PCR and gel electrophoresis and only those that tested positive were used for further experimentation. For this study, both male and female mice aged 1–6 months were used.

### Retinal Preparation

After euthanasia, the ventral side of the cornea was marked by a heat rod before enucleation. Eyes were enucleated and dissected in HEPES buffer solution composed of (in mM) 115 NaCl, 2.5 KCl, 2.5 CaCl_2_, 1.0 MgCl_2_, 10 HEPES, and 28 glucose, adjusted to pH 7.4 with NaOH. The dissection buffer was continuously bubbled with 100% oxygen during the procedure. The cauterization landmark was used to make a large incision in the ventral retina for the orientation of cardinal directions. Detailed dissection steps were previously described (Hellmer and Ichinose, 2015). Retinal preparations were kept in an oxygenated dark box until fixation. Whole retinas were fixed using incubation in 4% paraformaldehyde (PFA) in 0.1 M phosphate buffer (PB) for 1 h at room temperature. For transverse slice sections, retinas were fixed in 4% PFA for 30 min at room temperature. Both types of preparations were then washed with PB three times for 15 min each after fixation. Slice sections were then cryoprotected in 30% sucrose in PB overnight at 4°C. Samples were immersed in a 3:1 mixture of 30% sucrose in PB for 1 h at room temperature, embedded in 100% tissue freezing medium (Electron Microscopy Sciences #72592, Hatfield, PA, United States), and rapidly frozen in a dry ice/acetone bath. Slices were cut at 14 μm using a Cryotome cryostat (Thermo Fisher Scientific, Waltham, MA, United States) and dried in an oven at 55°C for 1 h.

### Immunostaining

Preparations were blocked for 1 h before staining by incubation at room temperature in 10% normal donkey serum (NDS) with 0.5% Triton X-100 in 0.01 M PBS (PBS-T). Primary antibodies were prepared in 3% NDS in PBS-T. Bipolar cells were identified by antibody staining. Anti-synaptotagmin-2 (Syt2) was used for type 2 bipolar cell soma staining and type 6 axon terminals, anti-hyperpolarization-activated cyclic-nucleotide gated channels (HCN4) for type 3a, anti-protein kinase A, regulatory subunit IIβ (PKARIIβ) for type 3b, anti-calsenilin (Csen) for type 4, and anti-protein kinase Cα (PKCα) for rod bipolar cells (RBCs) in accordance with previously characterized markers (Ghosh et al., 2004; Wässle et al., 2009). Starburst amacrine cells (SACs) were identified using antibodies against choline acetyltransferase (ChAT). Staining against s-opsin, highly expressed in the ventral retina, was used to validate the accuracy of the dissection marking method. A detailed list of primary antibodies can be found in Table 1. Slice preparations were incubated with primary antibody overnight at room temperature. Whole mounts were incubated with primary antibody for 48 h at 4°C. Phosphate-buffered saline (PBS) at a concentration of 0.01 M was used to wash the preparations three times for 15 min each. Secondary antibodies included donkey anti-rabbit Alexa 568, anti-mouse Alexa 568, anti-goat Alexa 633, and anti-mouse Alexa 647 (Thermo Fisher Scientific, Waltham, MA, United States). Secondary antibodies were dissolved in 3% NDS in PBS-T. Preparations were incubated with secondary antibody for 2 h at room temperature followed by washing in PBS three times for 15 min each. Preparations were placed on glass slides with ProLong Gold antifade reagent (Thermo Fisher Scientific) and a glass coverslip.

**TABLE 1:** Primary antibodies.

Antibody	Immunogen	Source, Cat.#, Species	RRID	Dilution
Calsenilin/DREAM, clone40A5	Full-length GST fusion protein of human Calsenilin	EMD Millipore, 05-756 Mouse monoclonal	AB_2313634	1:1,000
ChAT	Human placental enzyme	EMD Millipore, AB144P Goat monoclonal	AB_2079751	1:200
HCN4	GST fusion protein with amino acids 119-155 of human HCN4	Alomone Labs, APC-052, Rabbit polyclonal	AB_2039906	1:500
Opnsw1	Recombinant human blue opsin	EMD Millipore, AB5407, Rabbit polyclonal	AB_177457	1:100
PKARIIβ	Amino acids 1-418 of human PKARIIβ	BD Biosciences, 610625, Mouse monoclonal	AB_397957	1:3,000
PKCα	Amino acids 645-672 at C-terminus of human PKCα	Santa Cruz Biotechnology, sc-8393 Mouse monoclonal	AB_628142	1:500
Synaptotagmin-2	Zebrafish Syt2	Zebrafish International Resource Center, znp1, Mouse monoclonal	AB_10013783	1:200

### Imaging

Both slice preparations and whole mounts were imaged on a TCS SP8 confocal microscope (Leica, Germany) using an HC PL APO CS2 40 × water immersion objective. Slices were imaged at 1,024 × 512 pixels with a step size of 0.3 microns. For whole mounts, a total of 12 fields were imaged per retina, corresponding to four cardinal directions and three eccentricities each. Three eccentricities were defined as a center, approximately 500 μm away from the optic nerve head, middle, 1,000 μm away from the optic nerve head, and peripheral, 1,500 μm away from the optic nerve head. If the targeted region was free of damage, it was imaged. If damage or large vasculature was present, an adjacent field was imaged. Each field was imaged at 512 × 512 pixels and one-micron step size. The large ventral incision made during dissection was used for orientation, which was confirmed by the s-opsin staining (*n* = 1 eye). Each field was imaged as a *z*-stack encompassing the inner nuclear layer (INL) using GFP-labeled somas as a landmark. Central regions were noted as being in the central one-third of the retina near the optic nerve, middle regions the middle third, and peripheral regions the outer third.

### Image Analysis, Cell Counting, and Statistics

Colocalization analysis of GFP and bipolar cell subtype markers was performed using Leica Application Suite X imaging software (version 3.5.5., Leica). Cells were counted in 100 μm × 100 μm regions of interest. Counting of cells in the region of interest was performed independently by two to four people under the condition that the origin of tissue, region, and eccentricity were blinded. We used a manual cell counter plugin included with the Fiji distribution of ImageJ (Schindelin et al., 2012). We counted GFP-expressing bipolar cells only if the somas resided in the outer half of the INL. For antibody-stained cells, bipolar cells were determined and counted if their somas resided in the outer half of the INL and dendrites extending to the outer plexiform layer (OPL). All statistical analysis and graph preparation were performed using GraphPad Prism (version 9, GraphPad Software, La Jolla, CA, United States). Two-way ANOVA was used to analyze the bipolar cell topography data for orientation and eccentricity with Tukey’s multiple comparisons test. Linear regression was used to evaluate density gradients along the ventral-dorsal and nasal-temporal axes. Differences were considered significant if *p* < 0.05. SigmaPlot (version 14, Systat Software, San Jose, CA) was used to make color-coded heat maps using coordinates of counted fields and calculated densities.

## Results

### Two Strains of Mice Expressed Green Fluorescent Protein by Bipolar Cells in the Retina

**FIGURE 1:**
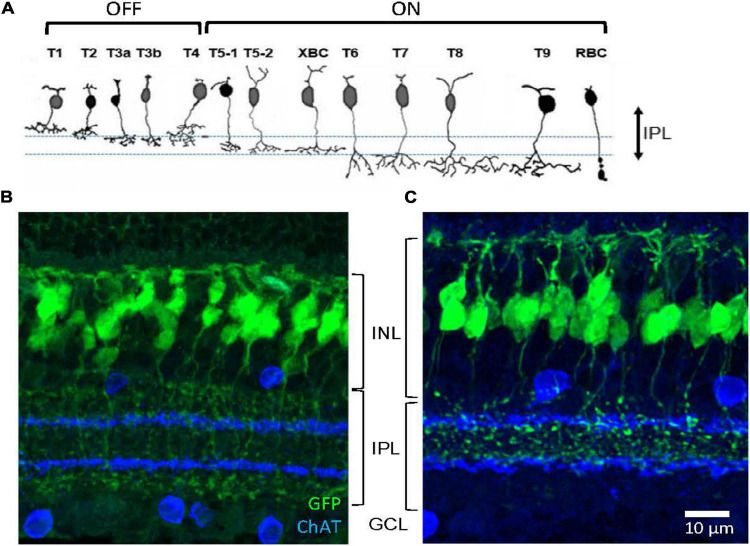
Two bipolar cell GFP strains. **(A)** Schematic showing 13 types of bipolar cells. Each type of cell ramifies differently on the OFF (upper) and ON ChAT bands (lower). **(B)** A transverse section shows GFP-expressing bipolar cells in the Rik mouse. Axon terminals of these cells ramified either outer to the OFF ChAT band or inner to the ON ChAT band in the IPL. **(C)** A transverse section shows GFP-expressing cells in the Lhx4 mice. Axon terminals ramified between two ChAT bands in the IPL. IPL, inner plexiform layer; INL, inner nuclear layer; GCL, ganglion cell layer.

First, we investigated the types of GFP-expressing cells in the Rik and Lhx4 mice, which were characterized as bipolar cell GFP strains (Siegert et al., 2009). For both strains, GFP-expressing somas were observed in the outer to the middle region of the INL, and processes extended both to the OPL and the inner plexiform layer (IPL) (Figure 1). After capturing the images in the slice preparations using a confocal microscope, we examined how the processes of each cell extended. A large majority of the cells that we examined were bipolar cells with dendrites extending to the OPL and axons extending toward the IPL (Rik, 511/575 cells, *n* = 5 mice; Lhx4, 524/551 cells, *n* = 4 mice), confirming that these are bipolar cell GFP mouse lines.

Approximately 15 subtypes of bipolar cells of the retina are identified in many species across the vertebrates, including humans, primates, rodents, fish, and salamander (Wu et al., 2000; Haverkamp et al., 2003; Connaughton et al., 2004; Ghosh et al., 2004; MacNeil et al., 2004). In the mouse retina, bipolar cell types have been characterized by their distinct patterns of axon terminal ramification in the IPL, particularly about the two cholinergic bands (Figure 1A; Ghosh et al., 2004; Ichinose et al., 2014; Ichinose and Hellmer, 2016). Axon terminals of the GFP-expressing bipolar cells in the Rik and Lhx4 mice ramified differently in the IPL (Figure 1). Bipolar cells in the Rik mice ramified either on the outer side of the OFF ChAT band or on the inner side of the ON ChAT band (Figure 1B), suggesting that these are types 1, 2, 6, 7, 8, 9, or rod bipolar cells. In contrast, bipolar cells in the Lhx4 mice ramified between two ChAT bands (Figure 1C), suggesting that these are type 3a, 3b, 4, or 5 bipolar cells. Bipolar cell types in the mouse retina are well investigated and their unique molecular expression has been revealed (Wässle et al., 2009; Shekhar et al., 2016). We used the type-specific antibodies to examine the bipolar cell types in these mouse lines.

**FIGURE 2:**
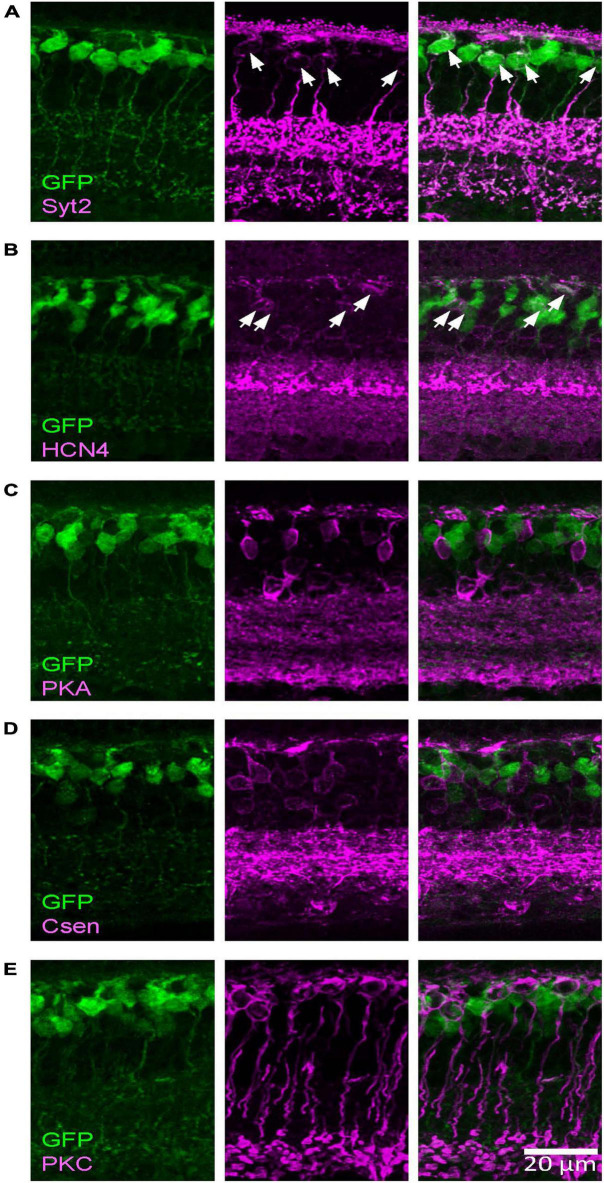
The Rik mouse GFP-expressing cells were type 2 and 3a bipolar cells. Images were taken from a single *z* section. **(A)** A type 2 bipolar cell marker, Syt2, colocalized with GFP-expressing cells. Asterisks indicate somas labeled both with Syt2 and GFP. **(B)** A type 3a marker, HCN4, colocalized with GFP cells. Asterisks indicate colocalized cells. **(C)** A type 3b marker, PKARIIβ, did not colocalize with GFP cells. **(D)** A type 4 marker, Csen, did not colocalize with GFP cells. **(E)** A rod bipolar cell marker, PKCα, did not colocalized with GFP cells.

By conducting immunohistochemistry, we labeled as type 2 (Syt2), type 3a (HCN4), type 3b (PKARII), type 4 (Csen), type 6 (Syt2), and rod bipolar cells (PKCα) and analyzed whether these cells colocalized with GFP-expressing bipolar cells. As shown in Figure 2, we found that Syt2-positive cells expressed GFP in the Rik mice (76/77 cell somas, 98.7% and 58/58 axon terminals in the sublaminae b, 100%). Notably, Syt2 labels type 2 cells from the soma to axon in the sublaminae a and the axon terminals of type 6 cells that ramify in sublaminae b. GFP-expressing axon terminals in the S1 IPL (outermost layer) and the S4–S5 IPL (innermost layer) completely colocalized with Syt2, indicating that GFP was expressed by type 2 and 6 bipolar cells and not by type 1, 7, 8, 9, or rod bipolar cells. Also, HCN4-positive cells expressed GFP (56/57 cells, 98.3%), indicating that the Rik mice expressed type 3a bipolar cells. However, GFP was not expressed by PKARIIβ-positive cells (0/23 cells), Csen (0/26 cells), and PKCα (0/92 cells). The number of cells was counted across 5 Rik mice. These results indicate that the GFP-expressing cells are a combination of type 2, 3a, and 6 bipolar cells.

**FIGURE 3:**
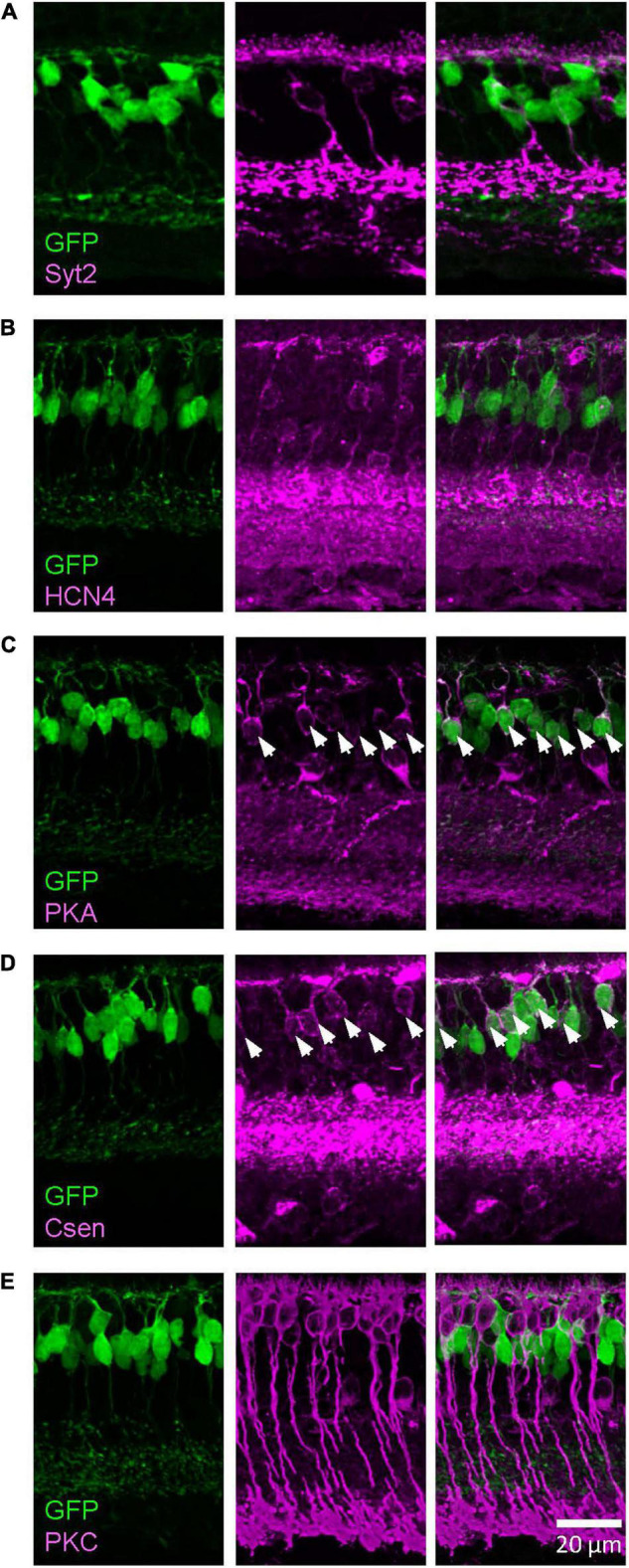
The Lhx4 mouse GFP-expressing cells were type 3b and 4 bipolar cells. Images were taken from a single *z* section. **(A)** A type 2 bipolar cell marker, Syt2, did not colocalize with GFP-expressing cells. **(B)** A type 3a marker, HCN4, did not colocalize with GFP cells. **(C)** A type 3b marker, PKARIIβ, colocalized with GFP cells, indicated by asterisks. **(D)** A type 4 marker, Csen, colocalized with GFP cells, indicated by asterisks. **(E)** A rod bipolar cell marker, PKCα, did not colocalize with GFP cells.

**FIGURE 4:**
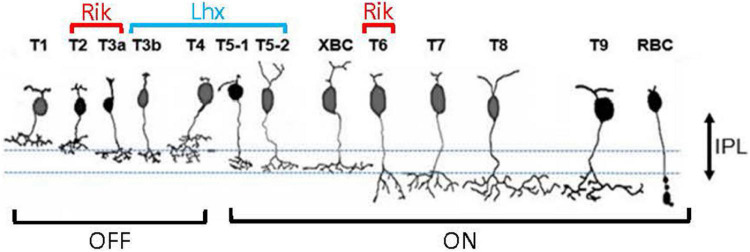
A summary panel showing bipolar cell types that are contained by the Rik and Lhx mice.

Similarly, we examined the GFP-expressing cells in the Lhx4 mice with the colocalization of markers (Figure 3). We found that PKARIIβ-positive cells expressed GFP in the Lhx4 mice (116/120 cells, 97%) and also Csen-positive cells (30/31 cells, 97%). GFP was not expressed by Syt2-positive cells (0/60 cells), HCN4 (0/34 cells), and PKCα (0/43 cells). The number of cells was counted across 4 Lhx4 mice. In addition, axon terminals of a subset of the GFP cells ramified near the ON ChAT band (Figure 1B), suggesting that they are type 5 ON bipolar cells (Hellmer et al., 2016). We did not observe the XBC, one of the type 5 bipolar cells with widely spread axon terminals (Helmstaedter et al., 2013; Hellmer et al., 2016). However, we could not distinguish other subsets. Therefore, GFP-expressing cells in the Lhx4 mice are a combination of type 3b, 4, and 5 bipolar cells. A summary of bipolar cell types expressing GFP in Rik and Lhx mice is shown in Figure 4.

### Topography of Green Fluorescent Protein-Expressing Cells in the Rik and Lhx4 Mice

Using these two mouse lines, we examined the distribution of bipolar cells in the wholemount retina. There is a heterogeneous distribution of distinct opsin-expressing photoreceptors on the retinal surface (Szel et al., 1992; Haverkamp et al., 2005; Peichl, 2005; Schiviz et al., 2008). The distinct distributions occur in the four retinal directions (ventral, dorsal, nasal, and temporal) and eccentricities from the optic nerve head. Therefore, we measured the density of GFP-expressing bipolar cells in three different eccentricities (central, intermediate, and peripheral retinas) in the four cardinal directions.

**FIGURE 5:**
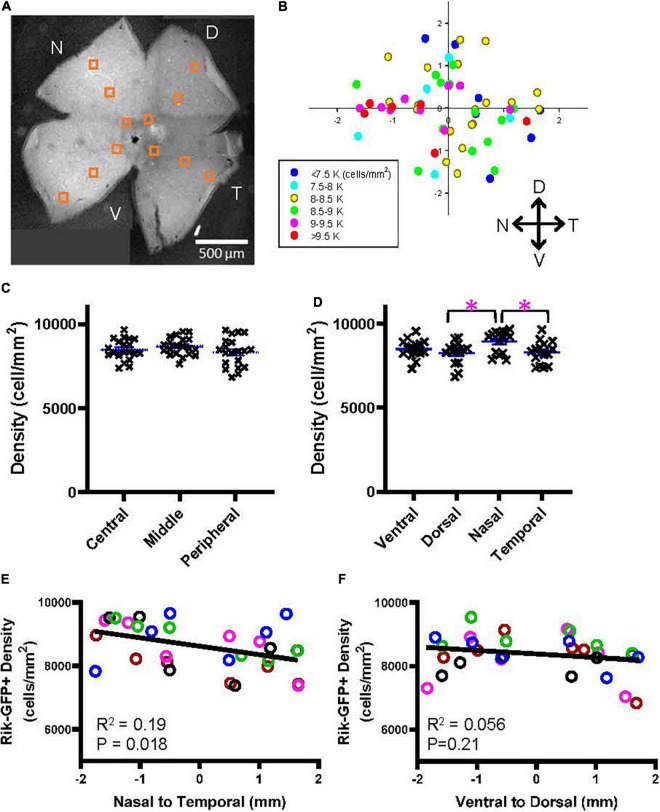
A heterogeneous distribution of GFP cells in the Rik mouse. **(A)** A wholemount retinal tissue from a Rik mouse eye. Boxes on the tissue indicate the region of interest (ROI), where cells were counted and analyzed. **(B)** A heatmap shows all ROI densities from 5 Rik retinas. GFP cells were highly distributed in the nasal region. **(C)** GFP cells at three eccentricities were plotted as a function of their density. Central, middle, and peripheral regions were defined by an approximate distance of 500, 100, and 1,500 μm away from the optic nerve head, respectively. No difference was observed between three groups. **(D)** All GFP cells were divided into four groups based on their cardinal directions. The cell density was significantly higher in the nasal direction than in the temporal and dorsal directions. Asterisks indicate statistical significance among the groups (*p* = 0.027: dorsal vs. nasal, *p* = 0.038: nasal vs. temporal, *n* = 5 retinas, 2-way ANOVA). **(E)** A linear regression analysis revealed that the GFP cell density was higher in the nasal direction along with the nasal-temporal axis. For this panel and all other linear regression analyses, data points from individual retinal tissues are color coded. **(F)** A linear regression analysis of GFP cell density along with the ventral-dorsal axis did not show uneven distributions.

Wholemount preparations were made from the Rik mouse eyes. After capturing cellular images using a confocal microscope, GFP-expressing cells were counted in 12 different locations (Figure 5A). On average, the density of GFP-expressing bipolar cells in the Rik mice was 8,486 ± 687 cells/mm^2^ (*n* = 5 retinas), which was comparable with the data suggested by Wässle et al. (2009) (total of type 2, 3a, and 6 was 8,457 cells/mm^2^). The cell densities for each region were plotted accordingly on the retinal locations (*n* = 5 retinas, Figure 5B), which suggested that cellular density was higher in the nasal region. We first examined the bipolar cell density as a function of the retinal eccentricity (Figure 5C), which revealed no difference among the distinct areas. Then, we analyzed the density as a function of the cardinal directions. We found that the cellular density was significantly higher in the nasal retina than in the temporal and dorsal retinas (Figure 5D, *p* = 0.038: nasal vs. temporal, *p* = 0.027: nasal vs. dorsal, *n* = 5 retinas, 2-way ANOVA). We also compared the densities along the nasal-temporal and the ventral-dorsal axes. The bipolar cell density in the Rik-GFP mice was higher in the nasal region along the nasal-temporal axis (*n* = 5 retinas, *R*^2^ = 0.19, *p* = 0.018) (Figure 5E). In contrast, the cellular density was not different along the ventral-dorsal axis (*R*^2^ = 0.06, *p* = 0.21) (Figure 5F). For these panels, data points from individual mice are color-coded, demonstrating the consistency across retinas.

**FIGURE 6:**
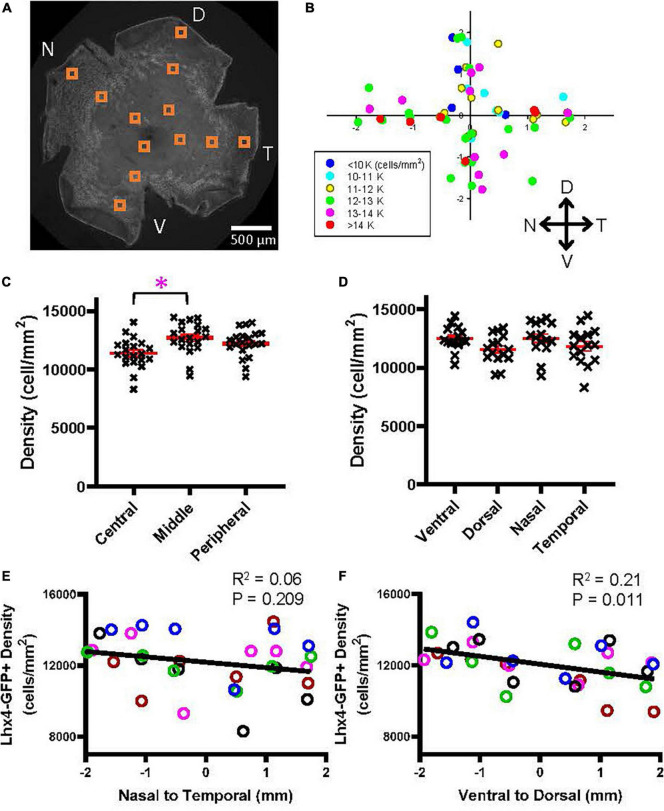
A heterogeneous distribution of the GFP cells in the Lhx4 mouse. **(A)** A wholemount retinal tissue from a Lhx4 mouse eye. Boxes on the tissue indicate the ROI, where cells were counted and analyzed. **(B)** A heatmap shows all ROI densities from 5 Lhx4 retinas. GFP cells were highly distributed in the ventral region. **(C)** GFP cells at three eccentricities were plotted as a function of their density. The middle eccentricity showed significantly higher cell density than the central retina (*p* = 0.005, *n* = 5 retinas, 2-way ANOVA). **(D)** All GFP cells were divided into four groups based on their cardinal directions. No significant difference in density was found between directions (2-way ANOVA). **(E)** A linear regression analysis revealed that the GFP cell density showed no difference along the nasal-temporal axis. An asterisk indicates statistical significance. **(F)** A linear regression analysis of GFP cell density along the ventral-dorsal axis showed a significant decrease going from the ventral to dorsal regions.

Similarly, the cellular density of the Lhx4 mouse eyes was measured (Figure 6). The overall average density of GFP-expressing bipolar cells in the Lhx mice was 12,100 ± 1,373 cells/mm^2^ (*n* = 5 retinas), which was comparable with the data suggested by Wässle et al. (2009) (total of type 3b, 4, and 5 was 11,260 cells/mm^2^). The density plot shown in Figure 6B suggested that GFP cells occurred at a higher density in the ventral and nasal regions. We first analyzed the bipolar cell density as a function of the eccentricity (Figure 6C), which revealed that the density was significantly lower in the central retina than in the middle retina (*p* = 0.005, *n* = 5 retinas, 2-way ANOVA). Then, we compared the density as a function of the four cardinal directions, which showed no significant effect (Figure 6D). The regression analysis revealed that there was no density difference along the nasal-temporal axis (*R*^2^ = 0.06, *p* = 0.20) (Figure 6E); however, the density in the ventral region was significantly higher along the ventral-dorsal axis (*R*^2^ = 0.21, *p* = 0.011) (Figure 6F). Taken together, results suggested that some types of bipolar cells exhibit a heterogeneous distribution along the retinal axes.

### Bipolar Cell Type-Dependent Asymmetric Distributions

Asymmetric distribution of OFF bipolar cells in the retina has been shown by Camerino et al. (2021). They reported that five types of bipolar cells occurred at a higher density in the ventral retina than in the dorsal retina. The bipolar cells in the Lhx4 mice, containing type 3b and 4 OFF bipolar cells, showed similar distribution patterns. However, cells in the Rik mice, containing type 2 and 3a OFF and type 6 ON bipolar cells, occurred at a higher density in the nasal region, which is not consistent with their findings. Therefore, we examined the distribution of each bipolar cell type in the Rik mice using the type-specific markers.

**FIGURE 7:**
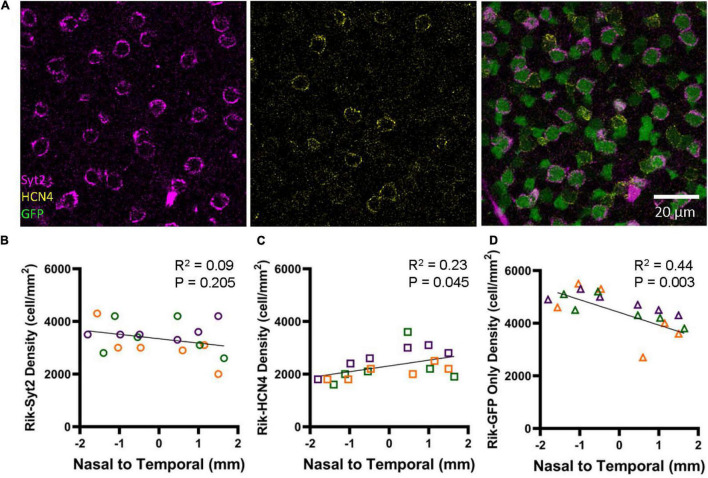
Bipolar cell type-dependent asymmetricity in the Rik mouse. **(A)** Subsets of GFP cells were colocalized with Syt2 and HCN4 antibodies, which revealed three different types of bipolar cells in the wholemount retinal tissues. **(B)** The density of Syt2-positive GFP cells did not show asymmetric distributions along the nasal-temporal axis (linear regression analysis). Each color on the graphs in B-D represent a different mouse. **(C)** The density of HCN4-positive, type 3a cells was higher along with the nasal-temporal axis (linear regression analysis). **(D)** The density of GFP+/Syt2-/ HCN4-, type 6 bipolar cells, was found to show a gradient of decreasing density moving from the nasal region to the temporal region (linear regression analysis).

Immunohistochemistry was conducted using the type 2 (Syt2) and 3a (HCN4) markers using three retinas representing three different Rik mice (Figure 7A). We analyzed 6 regions in each retina along the nasal-temporal axis. Bipolar cells that showed colocalization with antibody staining were counted; GFP showed high rates of colocalization with both Syt2 (602/607 cells, 99.2%) and HCN4 (416/422 cells, 98.6%). The distribution of Syt2-positive, type 2 bipolar cells is similar along the nasal-temporal axis (Figure 7B), and no difference was observed between temporal and nasal regions (Figure 7B, *n* = 3 retinas, *p* = 0.205, linear regression analysis). Interestingly, HCN4-positive, type 3a cells occurred at a higher density in the temporal region than in the nasal regions (Figure 7C, *n* = 3 retinas, *R*^2^ = 0.23, *p* = 0.045, linear regression analysis). Furthermore, we analyzed GFP-only cells that were neither labeled with Syt2 nor HCN4 and were type 6 ON bipolar cells (GFP + /Syt2-/HCN4-) (Figure 2). Notably, type 6 bipolar cells are positive with Syt2 only at the axon terminals but negative at the soma (Figure 2; Wässle et al., 2009). The GFP + /Syt2-/HCN4- cells occurred at a higher density in the nasal region along with the nasal-temporal axis (Figure 7D, *n* = 3 retinas, *R*^2^ = 0.44, *p* = 0.003, linear regression analysis).

Figure 5D shows that bipolar cell density is higher in the nasal retina than in the temporal retina. On average, the GFP cell density was 640 cells/mm^2^ higher in the nasal region (Figure 5). Figure 7 shows that HCN4 cell density was slightly higher in the temporal regions by 550 cells/mm^2^, on average. However, GFP + /Syt2-/HCN4- cell density was 1,030 cells/mm^2^ higher in the nasal region. Therefore, the net increase in the nasal regions was approximately 500 cells/mm^2^, which may explain the overall GFP staining results (Figure 5D). In conclusion, we found that type 6 bipolar cells occur at a higher density in the nasal retina than in the temporal retina. In contrast, type 3a cell density was higher in the temporal region.

## Discussion

### Summary of Results

We investigated the two GFP mouse lines and found that GFP was expressed by a unique set of achromatic bipolar cells. We found that GFP was expressed by type 2, 3a, and 6 bipolar cells in the Rik mice, whereas GFP was expressed by type 3b, 4, and 5 bipolar cells in the Lhx4 mice. Then, we examined the distributions of these bipolar cells in the retina. We found that GFP-expressing bipolar cells in the Rik mice occurred at a higher density in the nasal retina, whereas GFP cells in the Lhx4 mice occurred at a higher density in the ventral retina. It has been reported that OFF bipolar cells occur at a higher density in the ventral region, which explained the Lhx4 mice results. However, the Rik mouse distribution pattern was not consistent with their finding. Therefore, we used type-specific antibodies to examine which types contributed to the asymmetric distributions in the Rik mouse. We found that the density of HCN4-labeled type 3a cells was higher in the temporal region. Also, GFP + /Syt2-/ HCN4-, type 6 bipolar cells occurred at a higher density in the nasal region.

### Physiological Significance of Each Type of Bipolar Cell

Bipolar cells are second-order neurons that receive visual signaling from photoreceptors, which initiate multiple, parallel processing pathways (Wässle, 2004; Euler et al., 2014). Approximately 15 types of bipolar cells have been identified in the vertebrate retina, from fish and amphibians to mammals and primates (Wu et al., 2000; Haverkamp et al., 2003; Connaughton et al., 2004; Ghosh et al., 2004; MacNeil et al., 2004), which are thought to encode distinct components of image signals to support parallel processing. Among the 15 types of bipolar cells in the mouse retina, unique functions of some bipolar cells have been characterized; rod bipolar cells are the only type of bipolar cells mediating rod signaling. Other bipolar cells from type 1 through type 9 transmit cone-mediated signaling. Chromatic bipolar cells, also identified as type 9 bipolar cells, exclusively transmit UV-cone signaling (Haverkamp et al., 2005; Breuninger et al., 2011), and type 1 bipolar cells transmit M-cone signaling (Breuninger et al., 2011).

Type 2 through type 8 are achromatic cone bipolar cells. Their unique molecular expression has been reported (Wässle et al., 2009; Shekhar et al., 2016), indicating their unique roles in visual signaling. Although their distinct temporal processing has been demonstrated (Baden et al., 2013; Borghuis et al., 2013; Ichinose et al., 2014; Ichinose and Hellmer, 2016), unique roles of each type in image processing have not been understood. Because bipolar cells provide synaptic inputs to the third-order neurons, recent connectomic studies offer some clues.

Connectomic reconstruction of bipolar cells using serial block-face electron microscopy (SBEM) was first reported by Helmstaedter et al. (2013). They found that each type of bipolar cells exhibited distinct connectivity to SACs and to direction-selective ganglion cells (DSGCs), which are key neurons for motion detection (Euler et al., 2002; Lee and Zhou, 2006). Detailed connectivity of the SACs of bipolar cells was further revealed (Kim et al., 2014; Ding et al., 2016; Greene et al., 2016). These reports similarly found that type 2 and type 7 bipolar cells provide synaptic inputs at proximal dendrites of OFF and ON SACs, respectively, whereas type 3 and type 5 cells connect at more distal portions of OFF and ON SAC dendrites. DSGCs receive synaptic inputs directly from type 3, 4, and 5 bipolar cells (Helmstaedter et al., 2013; Yonehara et al., 2013). Collectively, these reports indicate that type 2, 3, 4, 5, and 7 bipolar cells play a role in motion detection.

Type 6 bipolar cells show unique longitudinally extended axon terminals (Figure 1A), and they are not primarily involved in the motion detection circuits. Instead, their roles in rod signaling have been reported. AII amacrine cells convey rod signaling, which receives synaptic inputs from rod bipolar cells and transmits the signal to ON cone bipolar cells through gap junctions. Although all types of ON cone bipolar cells are thought to have couplings, type 6 bipolar cells have a significantly large area of gap junctions with AII amacrine cells (Tsukamoto and Omi, 2017). Furthermore, type 6 bipolar cells provide inputs to dopaminergic amacrine cells *via* ectopic processes in the OFF sublamina of the IPL (Dumitrescu et al., 2009). These facts suggest that type 6 bipolar cells are one of the crucial components in light adaptation.

### Implication of Our Results

We used two GFP mouse strains to examine the distribution of bipolar cells in the different regions of the retina. We found that these strains contain GFP-expressing bipolar cells, which are thought to be achromatic types (Haverkamp et al., 2005; Breuninger et al., 2011). The GFP expression occurred almost entirely for the particular bipolar cell types (>98%, “Results” section), demonstrating that these mouse lines can be new bipolar cell markers. The distributions of these achromatic bipolar cells were relatively homogeneous compared with the topographic separation of two distinct opsin-expressing cones (Szel et al., 1992; Haverkamp et al., 2005). However, we found heterogeneity in the distributions of type 3a and 6 bipolar cells.

We found that type 3a bipolar cells occurred at a higher density in the temporal retina (Figure 7). Axon terminals of type 3 cells ramify in the OFF ChAT band, which are thought to be one of the key players for motion detection. When a mouse walks forward, the scenery runs backward, which would activate direction-selective neurons for the posterior toward the temporal direction. DSGCs for the posterior direction were genetically identified as DRD4 and TRHR ganglion cells (Rivlin-Etzion et al., 2011). According to the report provided by Rivlin-Etzion et al. (2011) TRHR-marked ON-OFF ganglion cells were slightly higher in the temporal retinal region. This may relate to our observation of the heterogeneous distribution of type 3a bipolar cells. Type 5 bipolar cells are similarly important for motion detection. However, in the Lhx4 mice, we did not observe the differential distribution of GFP cells along the nasal-temporal axis. Type 5 bipolar cells consist of multiple subsets (Helmstaedter et al., 2013; Hellmer et al., 2016), and not all subsets might play a role in motion detection. Therefore, unlike the type 3a cells in the Rik mice, type 5 cells in the Lhx4 mice did not contribute to the heterogeneous distribution along the temporal-nasal axis.

We also found that type 6 bipolar cells occurred at a higher density in the nasal region than in the temporal region (Figure 7). As mentioned above, type 6 bipolar cells are crucial for light adaptation rather than motion detection. However, light adaptation does not seem to require heterogeneity of their responsible networks. Rather than their functional requirement, it might relate to the cone expression, which occurs at a higher distribution in the nasal-ventral region (Ortin-Martinez et al., 2014).

## Conclusion

We used two bipolar cell GFP mouse strains to examine the distributions of achromatic bipolar cells. The heterogeneous distribution occurred in the axes of ventral-dorsal or nasal-temporal rather than the eccentricity (e.g., Figures 5C, 6C). Even though the distribution of bipolar cells was more homogeneous than the distribution of photoreceptors, we found the differential distribution of a couple of bipolar cell types along with the nasal-temporal axis. We will need further investigation of the functional significance of the heterogeneity.

## Data Availability Statement

The original contributions presented in the study are included in the article/supplementary material, further inquiries can be directed to the corresponding author/s.

## Ethics Statement

The animal study was reviewed and approved by the Institutional Animal Care and Use Committee at Wayne State University (protocol #20-10-2909).

## Author Contributions

TI designed the study. ZS and AS collected experimental data and performed statistical analysis. ZS and TI wrote the manuscript. All authors read, revised, and approved the submitted version of the text.

## Conflict of Interest

The authors declare that the research was conducted in the absence of any commercial or financial relationships that could be construed as a potential conflict of interest.

## Publisher’s Note

All claims expressed in this article are solely those of the authors and do not necessarily represent those of their affiliated organizations, or those of the publisher, the editors and the reviewers. Any product that may be evaluated in this article, or claim that may be made by its manufacturer, is not guaranteed or endorsed by the publisher.
